# Percutaneous Coronary Intervention in a Patient Presenting With Inferior Myocardial Infarction and an Anomalous Left Main Artery Originating From the Right Coronary Sinus

**DOI:** 10.7759/cureus.54568

**Published:** 2024-02-20

**Authors:** Taulant Gishto, Leonard Simoni, Naltin Shuka, Arlind Dragoshi, Artan Goda

**Affiliations:** 1 Cardiovascular Disease, University Hospital Center "Mother Teresa", Tirana, ALB; 2 Cardiovascular Medicine, University Hospital Center "Mother Teresa", Tirana, ALB

**Keywords:** cannulation, guide catheter, percutaneous coronary intervention, "anomalous coronary artery" "left anterior descending artery origion from rca", non-st segment elevation myocardial infarction (nstemi)

## Abstract

We present a case of a patient with inferior myocardial infarction (MI) and anomalous left main artery originating from the right coronary sinus. The left main artery and right coronary artery originated from the right coronary sinus but with separate ostia. The patient underwent revascularization of the right coronary artery with balloon angioplasty and a drug-eluting stent. Despite being rare, these anomalies can be life-threatening depending on the course of the artery, and when atherosclerotic disease is present, a revascularization strategy can be challenging. Knowing the existence of the left main artery anomaly is important to choose the right guide catheter to achieve successful cannulation and decrease the risk of complications, radiation exposure, and contrast usage.

## Introduction

Coronary artery anomalies are discovered in less than 1% of angiography series. The most prevalent coronary anomaly among all patients receiving coronary angiography is the anomalous origin of left circumflex (LCx) coronary arteries from the right sinus of Valsava (0.41%) [1-4]. Other coronary artery anomalies include the left main artery (LMA) originating from the right sinus of Valsalva (from the right coronary artery (RCA) or a separate ostium; 0.02%), anterior and unusually high origin of the RCA (0.18%), RCA originating from the left sinus of Valsalva (0.13%), and coronary artery fistulas. In the case of the left main artery originating from the right sinus of Valsava, the anomalous artery may take different courses in approaching the left side of the heart [1,2]. Despite being rare, these anomalies can be life-threatening not only by themselves because of the course of the artery but also because of challenging revascularization procedures in the case of atherosclerotic disease.

## Case presentation

A 63-year-old male was admitted to the Cardiology Unit following an episode of chest pain and elevation of cardiac enzymes. The patient had a several-month history of retrosternal squeezing pain, spreading on both arms and occurring during exertion, and episodes of high blood pressure. Smoking history, hypertension, and age were some of his risk factors. On physical examination, the patient was alert, in good general condition, with normal vital signs (blood pressure 125/70, heart rate 75 bpm, SpO2 of 97%, and temperature 36.5 °C). The patient's pulmonary and cardiac examinations were normal.

**Figure 1:**
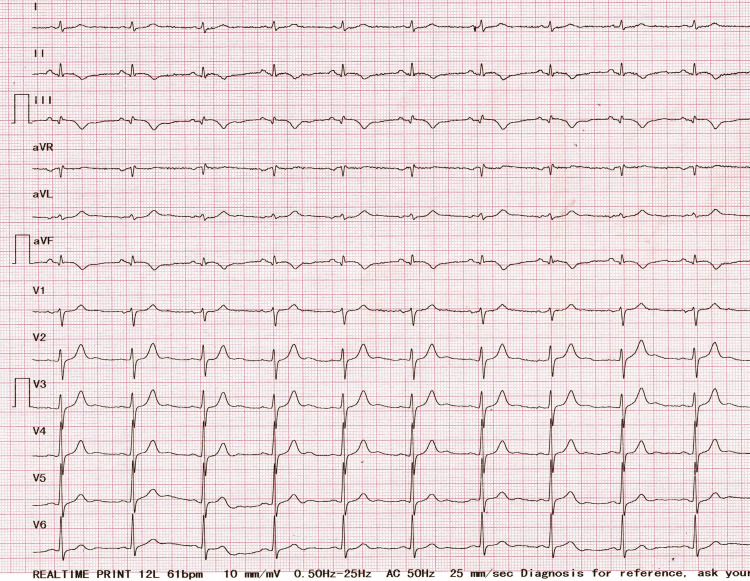
ECG of the patient on presentation Ischemic changes on inferior leads DII, DIII, aVF  (Q waves and negative T waves)

His resting ECG showed sinus rhythm, a heart rate of 75 bpm, slight ST elevation, and negative T waves on DII, DIII, and aVF (Figure 1).

On admission, the laboratory tests revealed an elevated Troponin I level of 0.170 ng/mL (normal range <0.034 ng/mL) 1 hour after the episode and increased to 0.530 ng/mL 6 hours from the episode. Complete blood count and biochemistry panel were in the normal range. The patient's echocardiography at admission demonstrated hypokinesis of basal inferior wall, normal size, and normal LV function (EF~0.58); mild left atrial dilation. Based on clinical evaluation, cardiac ultrasound, and laboratory tests, the patient was diagnosed with non-ST-elevation myocardial infarction (NSTEMI). An early reperfusion strategy was decided and he was referred to a catheterization laboratory to undergo coronary angiography. Coronary angiography revealed two-vessel coronary artery disease and anomalous origin of the LMA; LMA and RCA were both found to originate from the right coronary ostium. 

**Figure 2:**
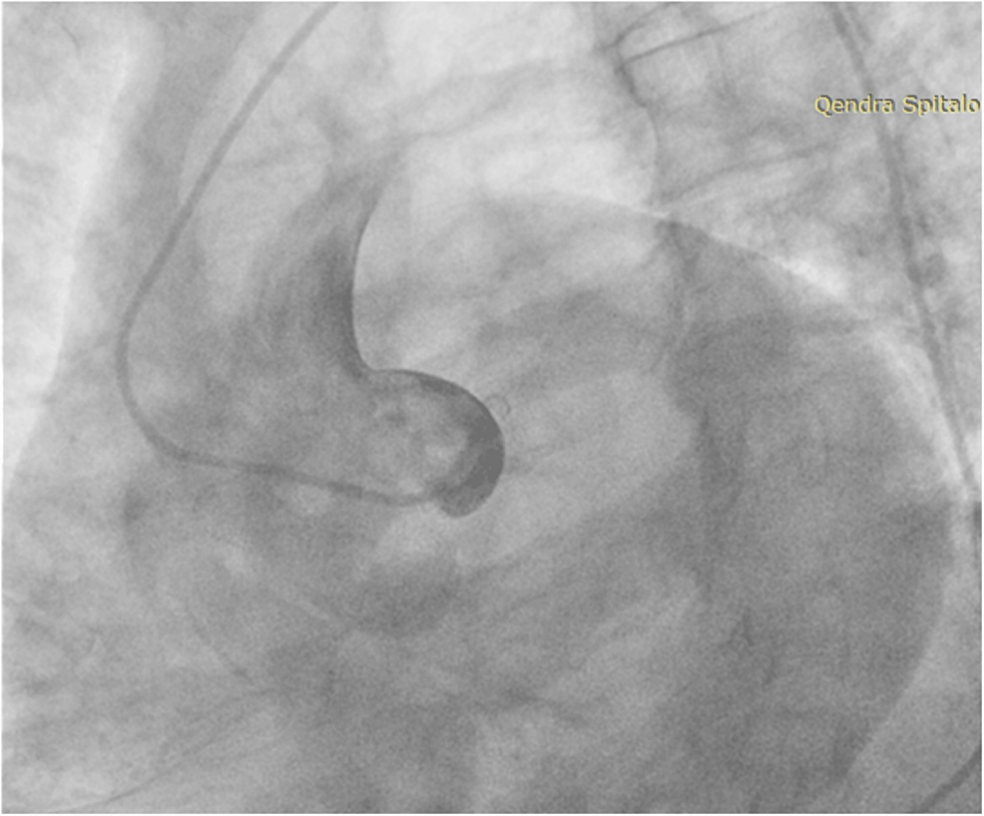
Attempts for Left Coronary Angiography Several attempts were made to cannulate the left main artery using different catheters but without success

**Figure 3:**
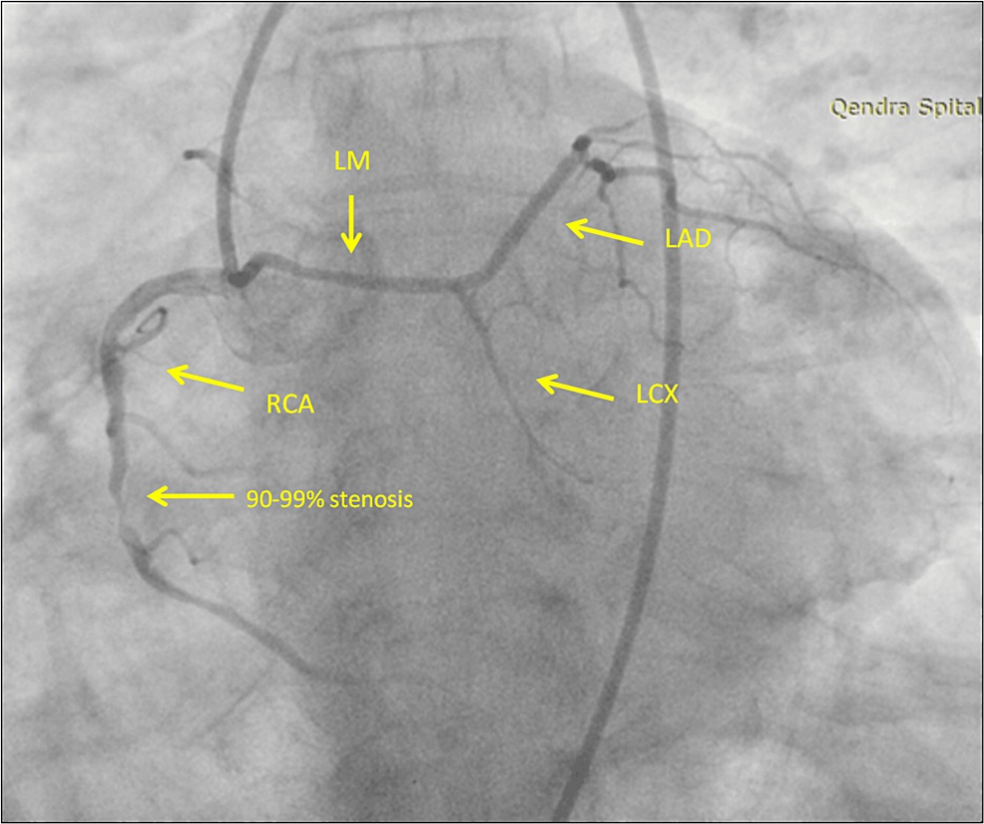
Anomalous origin of the left main artery. After many attempts, we decided to switch to a Judkins's Right catheter catheter to cannulate the right coronary artery (RCA), which showed the origin of the left main artery from the right coronary sinus. Different coronary angiography views demonstrated severe stenotic segments of the RCA, a 50% stenosis of the mid-left anterior descending artery (LAD), and a small normal left circumflex (LCX)

The initial attempt to cannulate the left coronary ostium to assess for left anterior descending and circumflex disease failed (Figure 2). We decided to shift to a Judkins's Right catheter to engage the right coronary ostium, which revealed a dominant RCA and aberrant origin of the LMA from the right coronary sinus. The aberrant LMA bifurcated into the left anterior descending artery (LAD) and a hypoplastic LCx (Figure 3).

There was a 99% stenotic lesion in mid-RCA and the Left Anterior Descending Artery (LAD) had a stable 50% stenosis on its mid-segment. Despite the anomalous course of the left main artery, the acute presentation of the patient was related to the RCA and unrelated to the aberrant vessel.

The challenging part of the revascularization strategy was partly choosing the correct guiding catheter to avoid ischemia of the left main artery during the procedure and mainly being careful during manipulation because any complication in the area of the right coronary ostium could cause fatal consequences to the patient.

**Figure 4:**
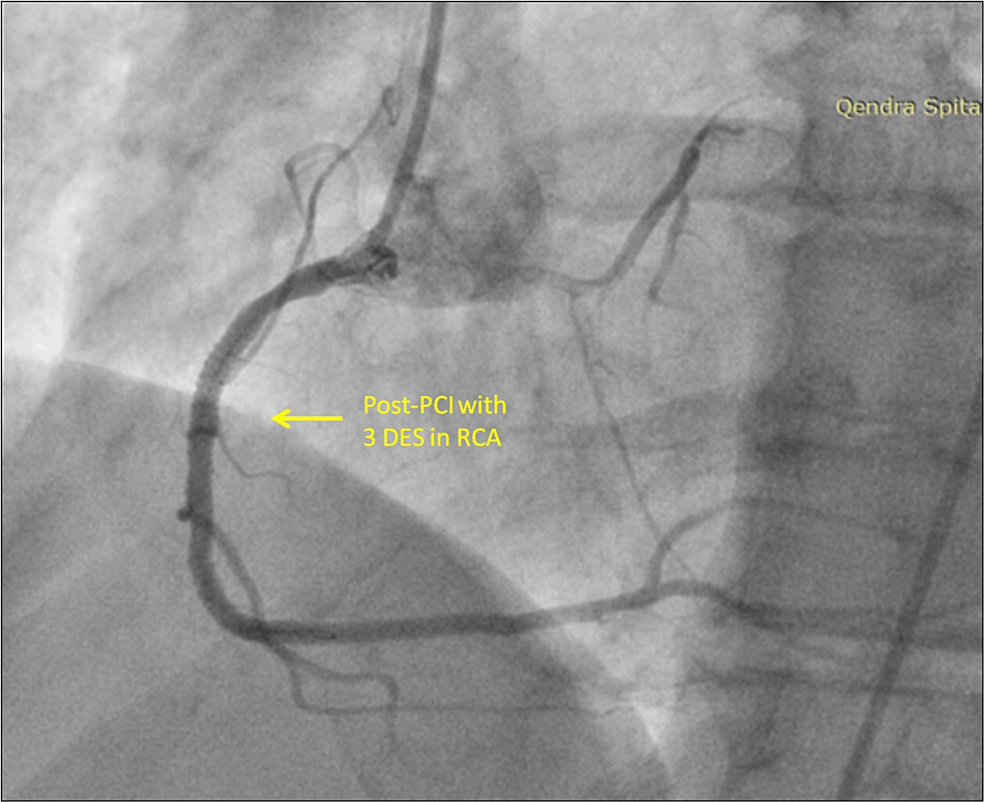
Revascularization procedure Predilatation of lesions was made using semi-compliant balloon catheters. Three drug-eluting stents (DES) were deployed successfully. Optimization of stent deployment using post-dilatation was made using non-compliant balloons. The final result was satisfactory with TIMI III flow and a perfect angiographic image of stents.

The patient underwent a challenging but successful revascularization of the RCA with balloon angioplasty and drug-eluting stents. Using a Judgkins Right 3.5 guiding catheter, the RCA lesion was predilated, and three drug-eluting stents (DES) were deployed successfully. The angiographic result was satisfactory, with TIMI III flow distally (Figure 4).

The case had a very challenging intervention because of the complexity of the coronary artery anatomy which required particular caution in avoiding right coronary ostium complications. The procedure was successful and without any setbacks. Two days later, the patient was discharged on guideline-directed medical therapy and in very good health.

## Discussion

Coronary artery anomalies occur in 0.3-0.9% of individuals without structural heart defects and in 3-36% of those with structural heart defects [1-2,4]. There are various coronary anomalies, however, occurrences of the LMA originating from the right coronary sinus are very rare (0.02%) [1-3]. An anomalous LMA originating from the opposite sinus may follow one of the following four courses: (1) anterior to the PA; (2) posterior to the aorta; (3) subpulmonic, also called septal (the anomalous coronary artery dives in the septum beneath the RVOT then re‐emerges more distally); (4) interarterial. The interarterial course is the one most associated with myocardial ischemia during exercise, with a subsequent risk of ventricular tachycardia (VT), ventricular fibrillation (VF), and sudden cardiac death [1,2,4]. The interarterial course is the most common course for an anomalous LMA. Various ideas have been proposed to explain the sudden cardiac deaths in these patients. The most widely recognized explanation for the increased frequency of ostium occlusion is that it results from compression between the major arteries during physical activity, creating a more slit-like orifice and occlusion [5].

A previous case report [6,7] presents a patient with monomorphic ventricular tachycardia as the first manifestation of an anomalous LMA which originated from the right sinus and had an interarterial course. Considering the anatomical variation of the anomaly and the episodes of ventricular tachycardia, surgical correction was chosen. Using an internal mammary artery graft, the LAD and LCx were bypassed, and the LMA was ligated successfully. A 2-year follow-up of the patient resulted in no new episodes of VT and withdrawal of medications.

Another case report [8] reported a patient with inferior wall myocardial infarction, and emergent cardiac catheterization revealed an anomalous LMA originating from the RCA as a single coronary artery. Angiography showed a large caliber RCA with a distal 99% stenotic ulcerated lesion, which was successfully predilated and stented. Shtembari et al. [9] have presented a case report similar to our case, where the LMA shared a single ostium with the RCA, and a lesion in the distal RCA was treated successfully with stents.

Defining the course taken by an artery in a two-dimensional projection like coronary angiography is not simple. Knowledge of the risk posed by anomalous arteris depending on their course is indispensable. We can precisely characterize the origin and course of each coronary artery with the help of modern imaging techniques like cardiac magnetic resonance (CMR) or coronary computed tomography angiography.

## Conclusions

Revascularization techniques should be based on the existence of obstructive coronary disease and the course of the anomalous LMA as this can favor one revascularization approach over the other. Further studies are needed to identify the best approach for these patients because of the complexity of the coronary anomalies presented. The main challenge during coronary intervention in a patient with a left main artery anomaly is choosing the right guide catheter to achieve successful cannulation and decrease the risk of complications, radiation exposure, and contrast usage.
